# Insulin-like growth factor-I induces chemoresistence to docetaxel by inhibiting miR-143 in human prostate cancer

**DOI:** 10.18632/oncotarget.22362

**Published:** 2017-11-09

**Authors:** Xiao-Bing Niu, Guang-Bo Fu, Lin Wang, Xin Ge, Wei-Tao Liu, Yi-Yang Wen, Hao-Ran Sun, Ling-Zhi Liu, Zeng-Jun Wang, Bing-Hua Jiang

**Affiliations:** ^1^ State Key Laboratory of Reproductive Medicine, Key Laboratory of Human Functional Genomics of Jiangsu Province, Jiangsu Key Laboratory of Cancer Biomarkers, Prevention, and Treatment Department of Pathology, Cancer Center, Nanjing Medical University, Nanjing, China; ^2^ Department of Urology, The First Affiliated Hospital of Nanjing Medical University, Nanjing, China; ^3^ Department of Urology, Huai’an First People’s Hospital, Nanjing Medical University, Huai’an, China; ^4^ Institute of Medical and Pharmaceutical Sciences, The Academy of Medical Sciences, Zhengzhou University, Zhengzhou, China; ^5^ Department of Pathology, Carver College of Medicine, The University of Iowa, Iowa City, IA, USA

**Keywords:** prostate cancer, docetaxel, miR-143, IGF-I, tumor growth

## Abstract

Elevated levels of insulin-like growth factor-I (IGF-I) are associated with carcinogenesis and cancer progression. However, the molecular mechanisms by which IGF-I promotes prostate cancer development remain to be elucidated. Docetaxel chemotherapy is an important therapeutic strategy in many types of human cancers including prostate cancer. In this study, we showed that IGF-I rendered PC-3 and DU145 cells more resistant to docetaxel treatment. IGF-I treatment decreased miR-143 expression, but increased the expression levels of IGF-I receptor (IGF-IR) and insulin receptor substrate 1 (IRS1), direct targets of miR-143. Overexpression of miR-143 abolished IGF-I-induced chemoresistance to docetaxel treatment, decreased expression levels of IGF-I, IRS1, and vascular endothelial growth factor (VEGF) in prostate cancer cell lines. Furthermore, docetaxel treatment significantly inhibited VEGF transcriptional activation, whereas IGF-I treatment induced VEGF transcriptional activation in a dose-dependent manner. Forced expression of IGF-IR and IRS1 cDNAs without the 3’ UTR regions restored miR-143-inhibited VEGF transcriptional activation. Finally, miR-143 inhibited tumor growth and made cells more sensitive to docetaxel treatment for decreasing tumor growth *in vivo*. Taken together, our data demonstrates that IGF-I induces docetaxel resistance and upregulates IGF-IR and IRS1 expression through miR-143 downregulation, whereas miR-143 acts as a tumor suppressor by targeting its targets IGF-IR and IRS1.

## INTRODUCTION

Prostate cancer (PC) is the second leading cause of cancer death among men in the United States and the fourth most common tumor type worldwide [1, 2]. Surgery, radiation and chemotherapy are generally effective for the majority of patients, but the prognosis of PC remains poor in patients with progressive disease. Docetaxel is widely used as a first-line drug for chemotherapy, but cell resistance to docetaxel is a hindrance for PC treatment. Thus, further understanding the molecular mechanism in promoting PC resistance to docetaxel is important and helpful for providing novel potential targets for PC treatment.

MicroRNAs (miRs) are a class of 19–22 nucleotide single-stranded small RNAs and act as major regulators of protein levels through either protein degradation or translational inhibition through binding to the 3’-UTR of target mRNA [3–6]. In human approximately 2,8645 unique mature miRNAs have been identified (http://mirdb.org/miRDB/). MiRNAs are known to be involved in a variety of biological processes, including development, differentiation, apoptosis, and cell proliferation [7]. As a result, miRNAs may function as either tumor suppressors or oncogenes. It has been demonstrated that miR-143 is a tumor suppressor in several types of cancer including prostate, breast and colorectal cancer [8–10]. To date, some genes including K-RAS, ELK1, IGF-IR, IRS1, Bcl-2 and ERK5 have been identified as direct targets of miR-143 [11–17]. In addition, by directly targeting protein-coding genes, miRNAs are capable to inhibit genes that are necessary for signaling pathways or drug-induced apoptosis to confer drug resistance. Up to now, multiple miRNAs have been found to be critical in the control of cancer drug resistance in PC. For example, downregulation of miR-205 and miR-31 promotes resistance by inhibiting chemotherapy-induced apoptosis in PC cells. It was reported that miR-148a attenuates paclitaxel resistance in PC3 cells by regulating MSK1 expression [18, 19].

Insulin-like growth factor (IGF) axis is one of the most investigated targets in cancer research due to the overexpression of its receptor in several types of human cancer including PC, and targeting IGF axis showed promising anti-tumor capabilities in preclinical studies [20]. In this study, we found that IGF-I rendered PC cells more resistant to docetaxel treatment. We also demonstrated that IGF-I downregulated miR-143 and upregulated IGF-IR and IRSI, the well-known oncogenes in carcinogenesis. Angiogenesis is important for tumor growth, angiogenesis, metastasis and drug resistance, and vascular endothelial growth factor (VEGF) is one of the strongest angiogenesis factors. Moreover, we demonstrated that miR-143 suppressed IGF-I-induced IGF-IR, IRS1, and VEGF expression and enhanced sensitivity to docetaxel treatment in the presence of IGF-I. Forced expression of IGF-IR and IRS1 restored miR-143-inhibited VEGF transcriptional activation. Finally, miR-143 inhibited tumor growth and increased sensitivity of PC to docetaxel and IGF-1 treatment *in vivo*. Our results reveal a novel mechanism of miR-143 in docetaxel resistance in PC, which is useful for developing new mechanism-based treatment option for PC in the future.

## RESULTS

### IGF-I promoted chemoresistance to docetaxel treatment in PC cells

**Figure 1:**
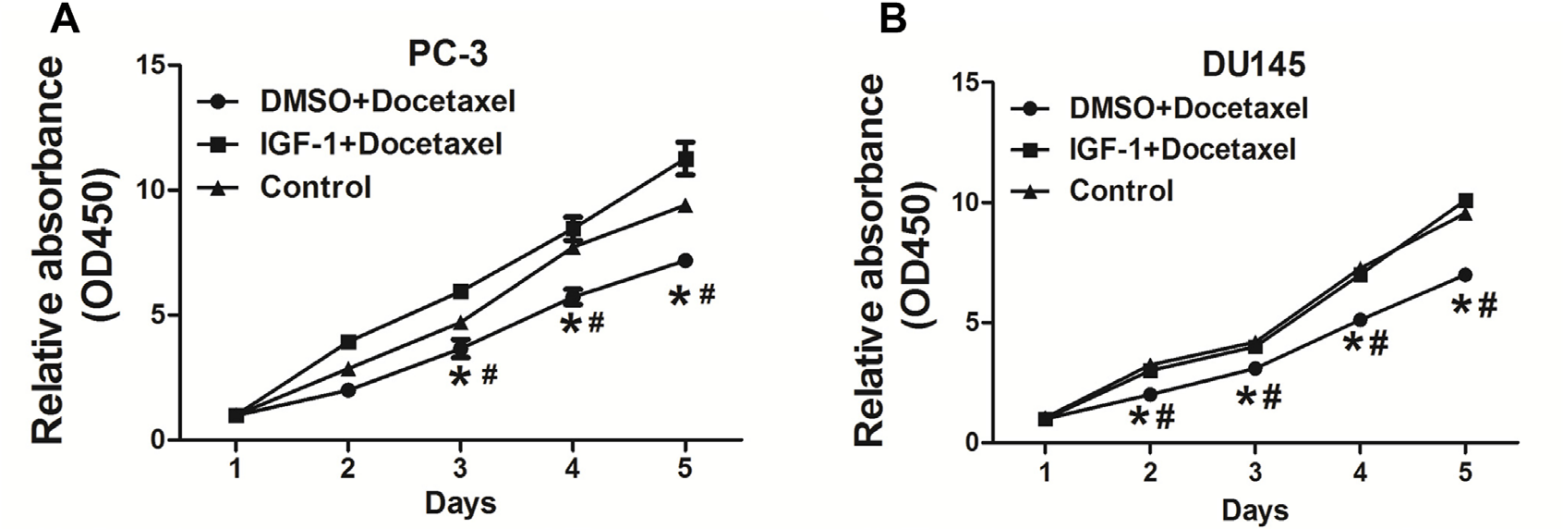
IGF-I promoted chemoresistance to docetaxel treatment in PC cells **(A)** PC-3 cells were treated with 10nM docetaxel, then cell proliferation was analyzed using CCK8 assay. The results showed that IGF-I promoted chemoresistance to docetaxel in PC-3 cells. **(B)** Similar experiments were performed using DU145 cells, and IGF-I also enhanced chemoresistance to docetaxel in DU145 cells. Data represent mean±SD. of three replicates. ^*^P<0.05 when compared to Control, ^#^*P*<0.05 when compared to IGF-I plus docetaxel treatment.

Docetaxel is used as a standard first-line drug for chemotherapy and is shown to have a survival advantage in metastatic castration-resistant PC (mCRPC) with two to three months of median survival advantage and improved life quality when compared to mitoxantrone treatment [21, 22]. Here we found that IGF-I increased resistance of PC-3 cells and DU145 cells to docetaxel treatment (Figure 1A and 1B), indicating that IGF-I axis may be involved in mediating docetaxel resistance in PC cells.

### IGF-I treatment induced IGF-IR and IRS1 expression in PC cells

**Figure 2:**
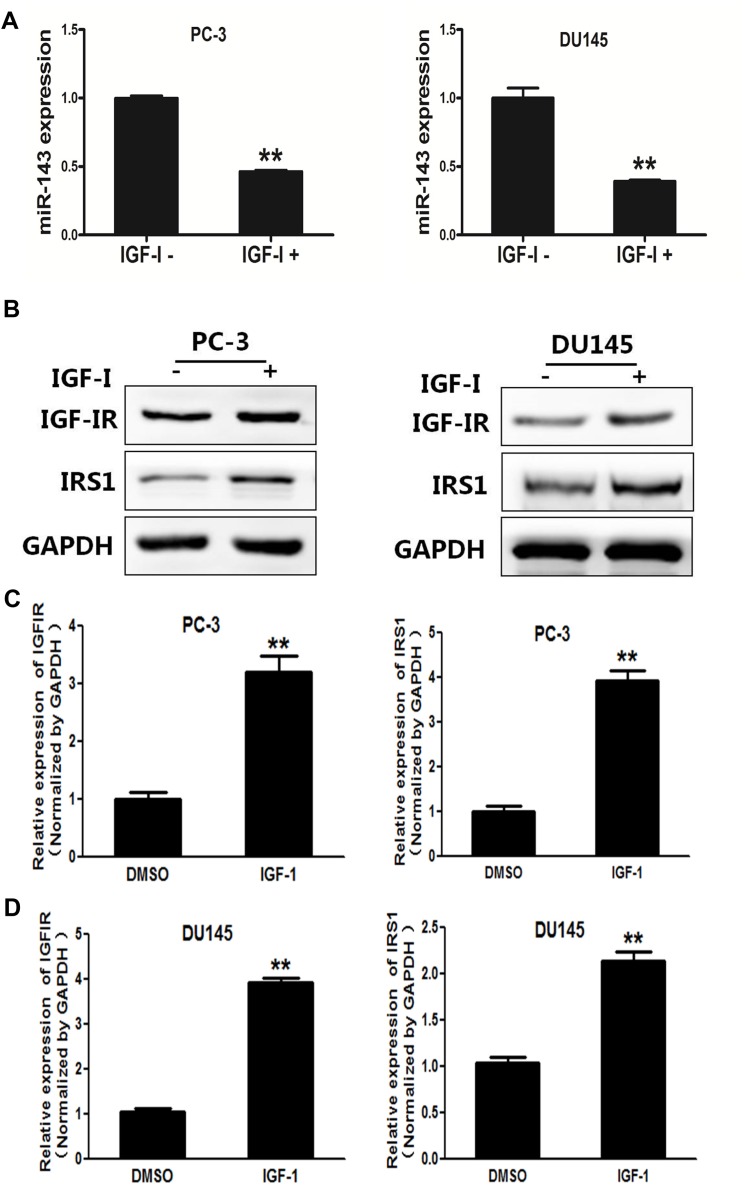
IGF-I induced expression levels of IGF-IR and IRS1 in prostate cancer cells **(A)** PC-3 cells were treated with IGF-I, then the expression of miR-143 was analyzed by RT-qPCR. The results showed that IGF-I treatment decreased miR-143 expression in PC-3 and DU145 cells. **(B)** PC-3 and DU145 cells were treated with IGF-I at 200 ng/ml or solvent control for 4 h, the expression of IGF-IR, IRS1 and GAPDH was determined using Western blotting. **(C)** PC-3 cells were treated with IGF-IR, and the mRNA levels of IGF-IR and IRS1 were analyzed using RT-qPCR and normalized by GAPDH expression. **(D)** Similar experiments were performed using DU145 cells treated with IGF-I. Data represent mean±SD. of three replicates. ^**^indicates significant difference at *P*<0.01.

Next, we found that IGF-I significantly decreased the expression levels of miR-143 in PC-3 and DU145 cells by 50% (Figure 2A). IGF-IR and IRS1 have been reported as direct targets of miR-143 [14, 15]. Consistent with the downregulation of miR-143, IGF-I treatment markedly induced expression of IGF-IR and IRS1 at both protein (Figure 2B) and mRNA levels in PC-3 and DU145 cells (Figure 2C and 2D). The activation of IGF-IR by binding to IGF-I leads to activation of numerous downstream pathways including PI3K/AKT and Raf/MAPK pathways. Insulin receptor substrate 1 (IRS1) is one of the main signal transmitters from IGF-IR to the downstream pathways [23]. Given the important role of IGF-IR and IRS1 in drug resistance [24, 25], these results suggest that miR-143 and its targets IGF-IR and IRS may be involved in IGF-I-induced docetaxel resistance in PC cells.

### MiR-143 suppressed IGF-I-induced chemoresistance to docetaxel treatment, and decreased expression of IGF-IR and IRS1, and VEGF transcriptional activation in PC cells

**Figure 3:**
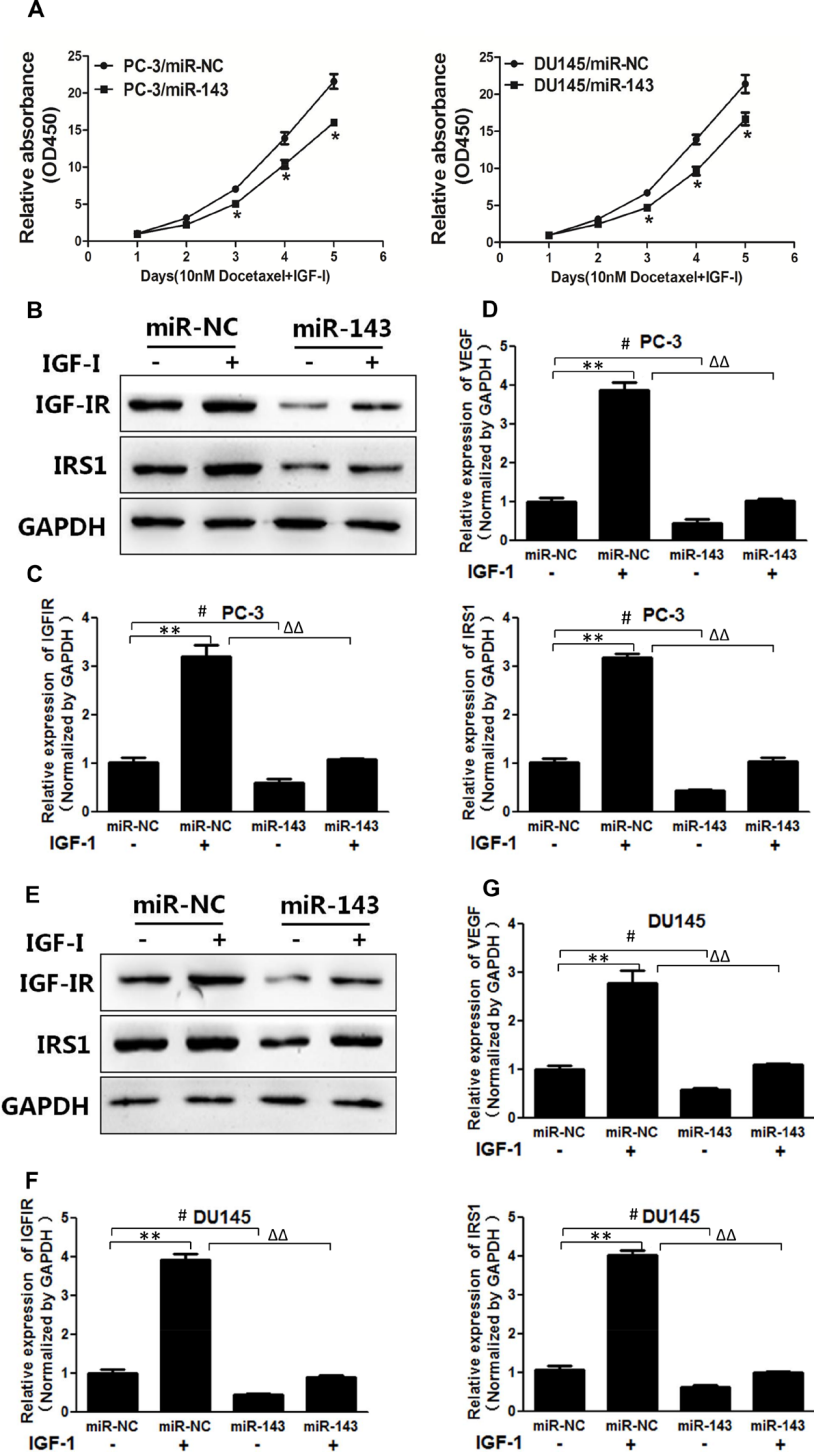
MiR-143 abolished IGF-I-induced IGF-IR, IRS1 expression, VEGF transcriptional activation and chemoresistance in PC cells **(A)** PC-3 and DU145 cells stably expressed with miR-143 or miR-NC were treated with IGF-I and docetaxel as above. Cell proliferation assay was performed using CCK8 assay. **(B)** PC-3 cells were transfected with miR-143 or miR-NC. Then the cells were treated with IGF-I or solvent control for 4 h. The expression of IGF-IR, IRS1 and GAPDH was assayed using Western blotting. **(C)** The expression levels of IGF-IR and IRS1 in cells treated as above were tested using RT-qPCR and normalized to the expression levels of GAPDH. **(D)** The expression levels of VEGF were tested using RT-qPCR and normalized to the expression levels of GAPDH. **(E-G)** DU145 cells overexpressing miR-143 and miR-NC were treated as above, and the expression of IGF-IR and IRS1 at protein and mRNA levels was determined using Western blotting and RT-qPCR assays as above. The expression of VEGF was also detected using RT-qPCR. Data represent mean±SD. of three replicates. ^**^P<0.01 when compared to IGF-I treatment without miR-143 overexpression; ^#^*P*<0.05 when compared to miR-143 overexpression without IGF-I Treatment; ^∆∆^*P*<0.01 when compared to miR-143 overexpression upon IGF-I treatment.

Our previous studies have demonstrated that miR-143 acts as a tumor suppressor to inhibit tumor growth, angiogenesis and increases the anti-cancer effects of oxaliplatin and temozolomide [14, 15, 26]. To further study the effect of miR-143 in regulating signal molecules, we found that miR-143 overexpression increased sensitivity to docetaxel treatment in PC cells in the presence of IGF-I (Figure 3A). IGF-I treatment upregulated IGF-IR and IRS1 expression in PC-3 cells, whereas overexpression of miR-143 inhibited IGF-I-induced IGF-IR and IRS1 expression (Figure 3B and 3C). VEGF is a vital angiogenesis factor during tumor angiogenesis, which exhibits an important role in tumor initiation, cancer development and chemoresistance [27, 28]. Forced expression of miR-143 also decreased IGF-I-enhancing VEGF mRNA levels in PC-3 cells (Figure 3D). Similar results were obtained using DU145 cells, showing that IGF-I increased IGF-IR, IRS1 and VEGF expression, and miR-143 attenuated protein levels of IGF-IR and IRS1 and mRNA levels of VEGF with or without IGF-I treatment (Figure 3E–3G), confirming the inhibitory effect of miR-143 on these signaling molecules. The results suggest that miR-143 enhances response to docetaxel treatment and reversed IGF-I-induced chemoresistance in PC cells.

### IGF-IR and IRS1 were downstream targets of miR-143 to inhibit VEGF transcriptional activation

**Figure 4:**
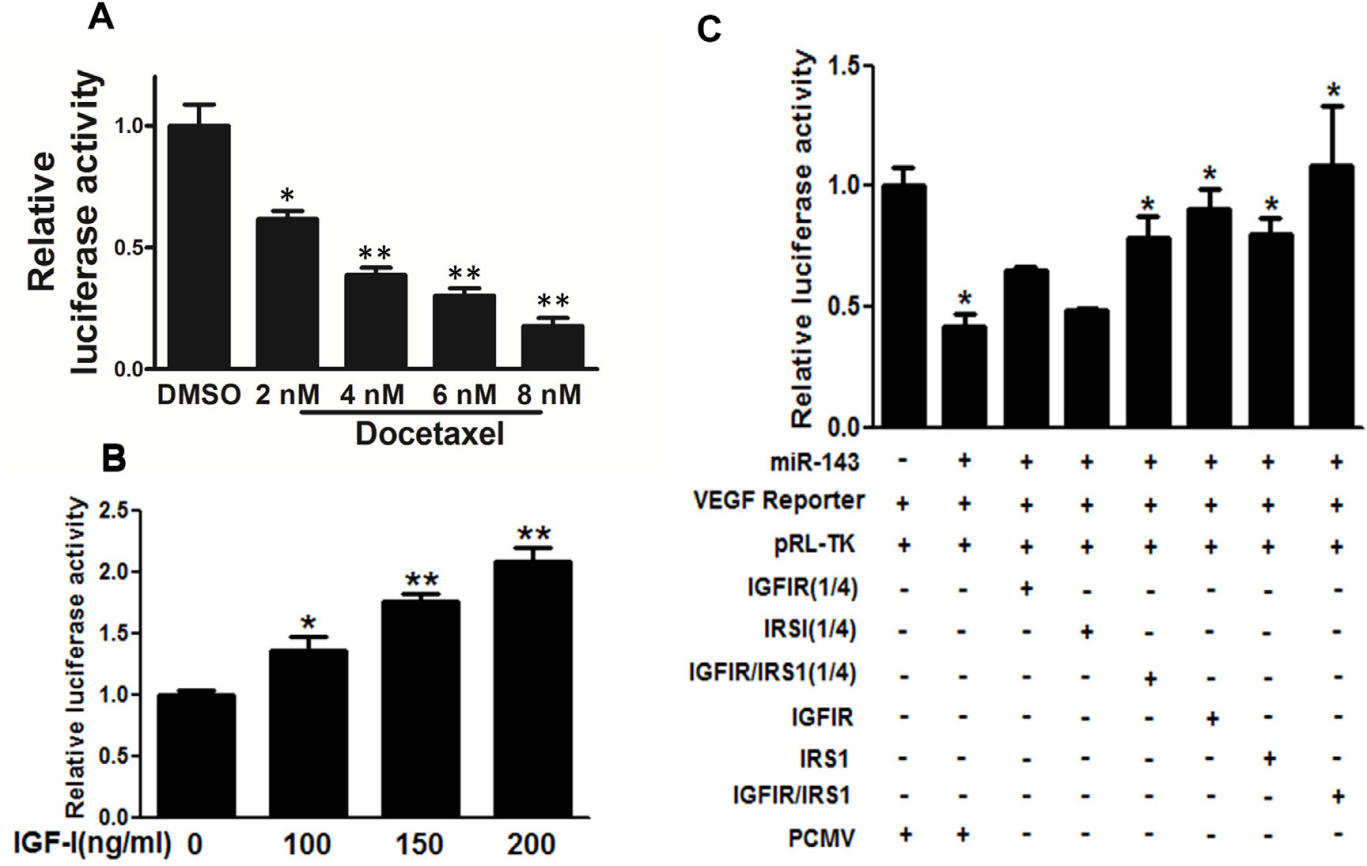
IGF-IR and IRS1 were two key downstream targets of miR-143 to inhibit VEGF transcriptional activation **(A)** PC-3 cells were treated with docetaxel at different concentration and cultured for 48 h, the transcriptional activation of VEGF was determined using luciferase assay. **(B)** PC-3 cells were treated with IGF-I at 200 ng/ml for 16 h and subjected to VEGF luciferase assay as above. **(C)** PC-3 cells stably expressing miR-143 or miR-NC were co-transfected with VEGF reporter, pRL-TK vector plasmid without or with IGF-IR or IRS1 cDNA plasmid at 50 ng (1/4 dose) or 200 ng (1 dose, without indication). The relative luciferase activities of VEGF reporter were assayed and calculated by the ratios of firefly/Renilla luciferase activities, which were normalized to that of the control. Data represent mean±SD. ^*^ indicates significant difference at *P*<0.05; ^**^ indicates significant difference at *P*<0.01.

VEGF is essential for endothelial cell function associated with angiogenesis. In this study, docetaxel treatment reduced transcriptional activity of VEGF in PC-3 cells (Figure 4A). IGF-I treatment increased VEGF transcriptional activity in PC-3 cells in a dose-dependent manner (Figure 4B). PC-3 cells stably expressing miR-143 or negative control were co-transfected with VEGF promoter reporter, pRL-TK vector plasmid without or with IGFIR or IRS1 cDNA plasmid at 50 ng (1/4 dose) or 200 ng (1 dose). The relative luciferase activities of VEGF reporter were assayed and calculated by the ratios of firefly/Renilla luciferase activities, which were normalized to that of the control (Figure 4C). The results showed that miR-143 overexpression decreased VEGF transcriptional activation to 45%, whereas forced expression of IGF-IR or IRS1 alone was sufficient to rescue miR-143-inhibiting VEGF transcriptional activity. Furthermore, the combination of IGF-IR and IRS1 at low concentration (1/4 dose each) significantly restored the VEGF transcriptional activation. The results demonstrate that IGF-IR and IRS1 are two key downstream mediators of miR-143 to attenuate VEGF transcriptional activation.

### MiR-143 inhibited tumor growth *in vivo*

**Figure 5:**
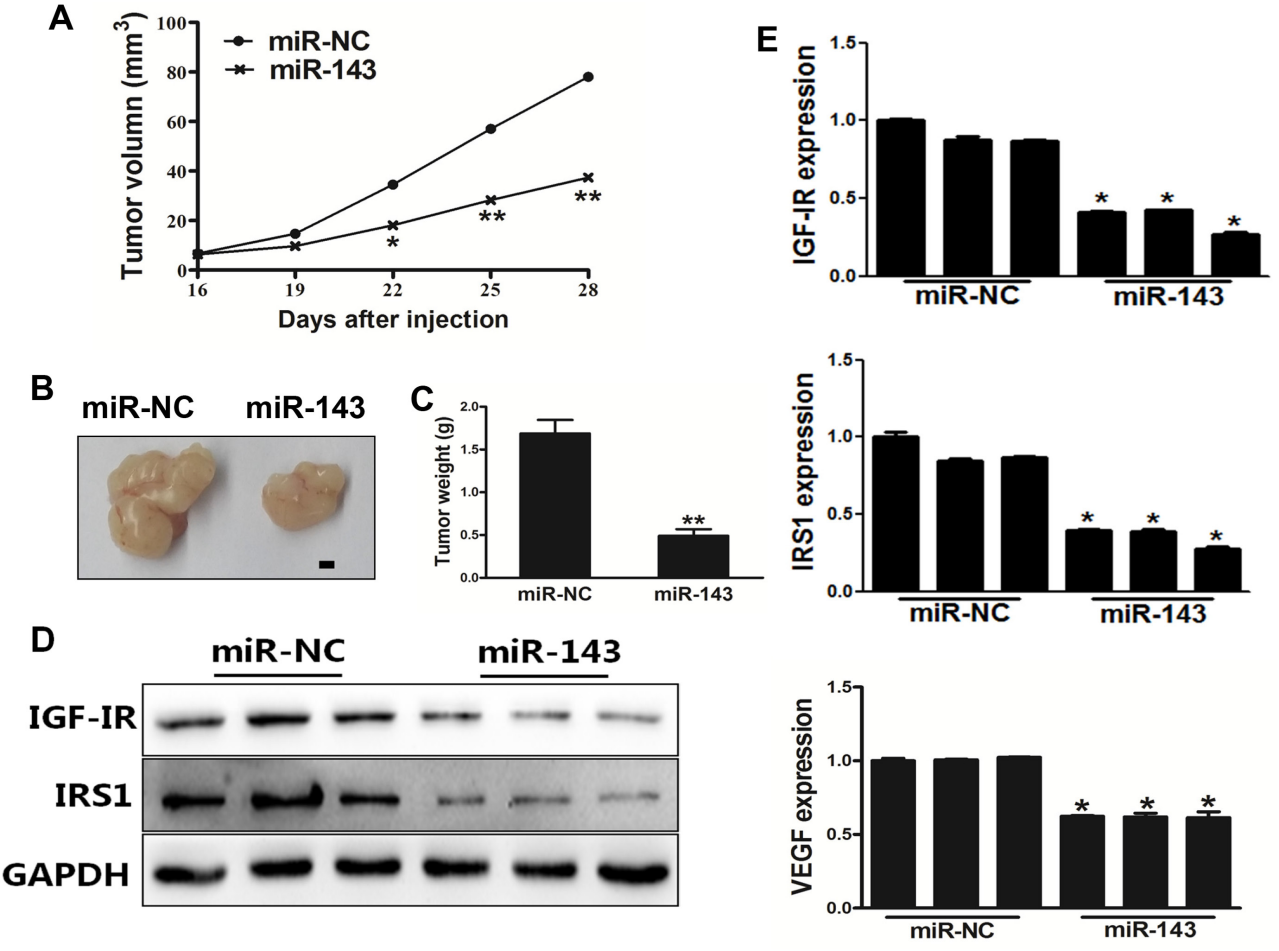
MiR-143 inhibited tumor growth *in vivo* **(A)** PC-3 cells were transduced with lentivirus carrying miR-143 or miR-NC and selected using puromycin. Cells stably expressing miR-143 or miR-NC at 5x10^6^ were subcutaneously injected into both flanks of male BALB/cA nude mice. Tumor volumes were monitored over time as indicated. **(B)** After 28 days post implantation, the xenografts were harvested and photographed. Representative pictures from each group were shown. Bar=2 mm. **(C)** The tumors from each group were weighed and the results showed miR-143 overexpression decreased tumor size and weight *in vivo*. **(D)** Total proteins were assayed by Western blotting to determine levels of IGF-IR and IRS1. Levels of GAPDH were used as internal control. **(E)** Total RNAs were isolated and assayed by qRT-PCR to determine the expression of IGF-IR, IRS1 and VEGF in tumors. Data represent mean±SD. ^*^ indicates significant difference at *P*<0.05; ^**^ indicates significant difference at *P*<0.01.

We next investigated the effect of miR-143 on tumor growth of PC-3 cells using nude mice. PC-3 cells stably expressing miR-NC or miR-143 were subcutaneously injected into male BALB/cA nude mice to study tumor growth. The results showed that miR-143 significantly attenuated tumor growth with decreased tumor size and weight (Figure 5A–5C). In agree with the results from *in vitro* experiments above, the expression levels of IGF-IR, IRS1 and VEGF from the tumor tissues of miR-143 expressing group were lower than those from miR-NC group (Figure 5D–5E), demonstrating that miR-143 acts as a tumor suppressor to inhibit tumor growth in PC cells, and IGF-IR and IRS1 are targets of miR-143 *in vivo*.

### MiR-143 sensitized PC cells to docetaxel treatment *in vivo*

**Figure 6:**
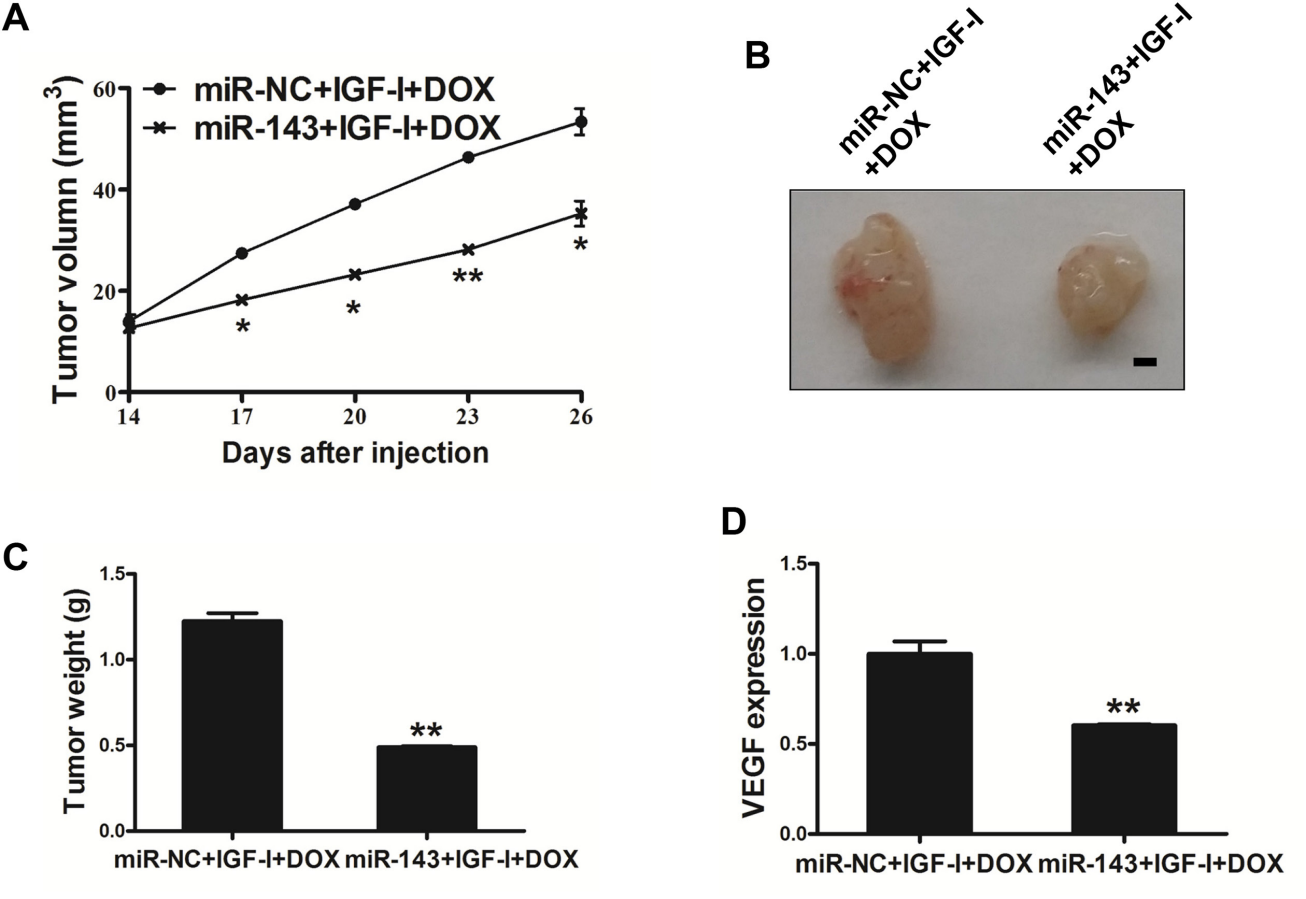
MiR-143 sensitized the PC cells to docetaxel treatment *in vivo* **(A)** PC-3 cells stably expressing miR-143 or miR-NC at 5x10^6^ cells were subcutaneously injected into both flanks of male BALB/cA nude mice. Tumor volume was monitored over time as indicated, then IGF-I (200 ng/ml) and docetaxel (10 nM) was administrated by peritoneal injection. **(B)** After 28 days post implantation, the xenografts were harvested and photographed. Representative pictures from each group were shown. Bar=2 mm. **(C)** The tumors from each group were weighed and the results showed miR-143 overexpression decreased tumor weight *in vivo*. **(D)** Total RNAs were isolated and assayed by RT-qPCR to determine the expression of VEGF in tumors. Data represent mean ± SD. ^*^ indicates significant difference at *P*<0.05; ^**^ indicates significant difference at *P*<0.01.

Finally, in order to study whether miR-143 sensitize PC cells to docetaxel and IGF-I treatment, PC-3 cells stably expressing miR-143 or miR-NC were subcutaneously injected into both flanks of male BALB/cA nude mice. IGF-I (200 ng/ml) and docetaxel (10 nM) was added 14 day later by peritoneal injection. After 28 days post implantation, the xenografts were harvested and photographed (Figure 6A–6B). The results showed that miR-143 overexpression further increased docetaxel effect for inhibiting tumor volume and weight *in vivo* (Figure 6A–6C). As expected, expression levels of VEGF in tumor tissues from miR-143 expressing group were significantly lower than those from miR-NC group (Figure 6D). Thus, these results indicate that miR-143 renders PC cells more sensitive to docetaxel treatment *in vivo*.

## DISCUSSION

PC is one of the most common cancers in males. However, the molecular mechanisms of PC progression and drug resistance are still to be uncovered. Growing evidence has shown that miRNAs are dysregulated and involved in cancer progression, and some of miRNAs may be potential targets in cancer diagnosis and treatment [29]. MiRNAs have shown multiple roles of in PC pathogenesis and are associated with the initiation and progression of PC [30, 31]. Among the identified tumor-suppressing miRNAs, miR-143 has been well studied in human PC [10, 32, 33]. Docetaxel is a common chemotherapeutic agent used to treat multiple malignancies including PC. However, there are no good biomarkers of response/resistance known for docetaxel treatment. Molecular profiling of miRNAs may be helpful for developing molecular diagnostics and identifying subgroups of patients destined to respond to docetaxel, and also for better understanding the molecular mechanisms of chemoresistance [34, 35].

Recently, several studies have demonstrated that IGF-I is associated with an increased risk of developing several types of cancers including lung, breast, colorectal, and PC [36–39]. IGF-IR and IRS1 have been reported as direct targets of miR-143. However, the role and underlying mechanism of miR-143 in mediating docetaxel resistance in PC remain elusive.

In the present study, we found that IGF-I- and its ligand IGF-IR-mediated signal molecules were often upregulated in PC, efficiently rendered PC-3 and DU145 cells more resistant to docetaxel treatment. IGF-I decreased miR-143 and increased its targets IGF-IR and IRS1 expression levels. Moreover, miR-143 inhibited VEGF transcriptional activation through IGF-IR and IRS1, and suppressed IGF-I-induced chemoresistance to docetaxel treatment. Finally, miR-143 inhibited tumor growth and increased sensitivity of PC to docetaxel and IGF-I treatment *in vivo*. In summary, we report here that IGF-I induces chemoresistence to docetaxel treatment in PC, and miR-143 and its targets IGF-IR and IRS1 are involved in this process.

## MATERIALS AND METHODS

### Cell culture and reagents

Human PC cells PC-3 and DU145 were maintained in RPMI1640 medium, and human embryonic kidney 293T (HEK-293T) cells were cultured in DMEM medium, supplemented with 10% fetal bovine serum, 100 units/ml penicillin, and 100 μg/ml streptomycin. Cells were cultured in humidified 5% CO_2_ incubator at 37°C. Recombinant human IGF-I were purchased from Sigma (St louis, MO, USA).

### Lentiviral packaging and stable cell line establishment

Lentivirus carrying miR-143 and miR-NC (negative control) were packaged in HEK-293T cells using lentiviral packaging kit following the manufacturer’s instructions (Open Biosystems, AL, USA). Stable cell lines PC-3/miR-143 and PC-3/miR-NC were established by lentiviral transduction in the presence of polybrene and screened by puromycin (Sigma, MI, USA).

### Cell proliferation assay

Cells were seeded in 96-well plates at confluence of 2000 cells per well. The capability of cell proliferation was measured using a CCK8 kit (Dojindo Laboratories, Kumamoto, Japan) according to the manufacturer’s instruction at different indicated time points. Data were from three separate experiments with six replications each time.

### RNA isolation and quantitative real-time PCR (qPCR)

Total RNAs of cells were extracted using TRIzol reagent (Invitrogen, CA, USA) according to the manufacturer’s instruction. To test miR-143 expression, expression of U6 was used as an endogenous control. To determine expression of miR-143 forward primer: GCTCGTCGAGATAAGCTGTGTG; reverse primer: GTTCGCTGAGATGAAGCACTG. To determine the mRNA levels of VEGF, total RNAs were reversely transcribed by oligodT primer using PrimeScript RT Reagent Kit (Vazyme, Nanjing, China). Housekeeping gene GAPDH was used as internal control. The cDNAs were amplified by qPCR using AceQ SYBR Master Mix (Vazyme, Nanjing, China) on a 7900HT system. Relative fold changes in expression of the target gene transcripts were determined using the comparative cycle threshold method (2^-ΔΔCt^).

### Protein extraction and western blotting

Cells or grounded tissues were lysed on ice for 30 min in RIPA buffer (150 mMNaCl, 100 mM Tris, pH 8.0, 0.1% SDS, 1% Triton X-100, 1% sodium deoxycholate, 5 mM EDTA and 10 mM NaF) supplemented with 1 mM sodium vanadate, 2 mM leupeptin, 2 mM aprotinin, 1 mM phenylmethylsulfonyl fluoride (PMSF), 1 mM DTT, and 2 mM pepstatin A. The lysates were centrifugated at 12,000 rpm 4 °C for 15 min, and the supernatants were collected. The protein concentration was determined using BCA assay (Beyotime Institute of Biotechnology, Jiangsu, China). Protein extracts were separated by SDS-polyacrylamide gel electrophoresis (SDS-PAGE), and transferred to nitrocellulose membranes in transfer buffer (20mM Tris, 150mM glycine and 20% (v/v) methanol). Membranes were blocked with 5% nonfat dry milk in 1× PBS containing 0.05% Tween-20 and incubated with antibodies against IGF-IR, IRS1 and GAPDH. Antibodies against IGF-IR and IRS1 were purchased from Cell Signaling Technology (Beverly, MA, USA). GAPDH antibody was from Bioworlde. The protein bands were probed with secondary antibody IgG conjugated to horseradish peroxidase, and visualized with the SuperSignal West Pico Chemiluminescent Substrate Kits (Thermo Scientific, MA, USA).

### Luciferase assays

For plasmid transient transfection, PC-3 cells stably expressing miR-143 or negative control were co-transfected with VEGF reporter [40], pRL-TK vector plasmid without (pCMV6 vector alone) or with pCMV6-IGF-IR or pCMV6-IRS1 plasmid (OriGene Technologies, Rockville, USA) at 50 ng (1/4 dose) or 200 ng (1 dose) using Lipofectamine 2000 (Invitrogen, Carlsbad, USA). Firefly luciferase (Luc) and Renilla luciferase activities were measured using a dual luciferase assay kit (Promega, Madison, USA). Each experiment was repeated three times with three replicates.

### Xenograft studies

For tumor growth assay, male nude mice [BALB/cA-nu (nu/nu), 6-week-old] were purchased from Shanghai Laboratory Animal Center (Chinese Academy of Sciences, Shanghai, China), and animals were maintained under special pathogen-free (SPF) conditions. Aliquots of cells (5×10^6^) were suspended in 150 μL of FBS-free RPMI 1640 medium and subcutaneously injected into each side of the posterior flank of nude mice. Tumor size was measured using vernier caliper every 2 days when they became visible, and the tumor volume was calculated according to the formula: Volume = 0.5 × Length × Width2. For chemoresistant effects of miR-143 *in vivo*, 14 days after implantation, IGF-I (200 ng/ml) and docetaxel (10 nM) were intraperitoneal injected into mice for docetaxel and IGF-I-treatment.

### Statistical analysis

All experiments were performed in triplicate, and data were analyzed with GraphPad Prism 5 (La Jolla, CA, USA). Statistical evaluation for data analysis was determined by student t-test. *P*<0.05 was considered as statistically significant.
